# Anticancer potential of fused heterocycles: structural insights and mechanistic advances

**DOI:** 10.2478/abm-2025-0035

**Published:** 2025-12-31

**Authors:** Aaysha Pandey, Shubham Sharma, Kamal Kishore, Swati Rani, Man Vir Singh, Gangotri Pemawat

**Affiliations:** School of Allied Sciences, Department of Chemistry, Dev Bhoomi Uttarakhand University, Dev Bhoomi Campus, Dehradun Uttarakhand 248007, India; Organic Synthesis Research Laboratory, Department of Chemistry, GLA University, Mathura, Uttar Pradesh 281406, India shubham.sharma@gla.ac.in; School of Civil, Mining, Environmental and Architectural Engineering, Department of Civil Engineering, University of Wollongong, Wollongong, New South Wales 2500, Australia; Department of Chemistry, Miranda House, University of Delhi, Delhi 110001, India; School of Allied and Life Science, UIT, Uttaranchal University, Dehradun 248007, India; Department of Chemistry, Mohanlal Sukhadia University, Udaipur, Rajasthan 313001, India

**Keywords:** *β-* lactam, anti-cancer, carbazole, isatin, pyrido[2,3-d]pyrimidine, pyrrolo-benzodiazepine

## Abstract

β-lactam derivatives, carbazoles, isatin derivatives, pyrrolo-benzodiazepines (PBDs), and pyrido[2,3-d]pyrimidines have demonstrated potential as anticancer agents among organic compounds. They exhibit substantial anticancer efficacy across several cancer cell lines, such as HL-60, THP-1, U-937, HeLa, PANC1, MDA-MB-231, and A549 cell lines. These compounds display a significant anticancer profile via diverse biological pathways such as DNA interaction, kinase inhibition, microtubule disruption, and enzyme inhibition. Their low IC50 values across various cell lines suggest their viability as strong candidates for targeted and multi-mechanistic cancer therapy, warranting further in vivo and clinical exploration. This review thoroughly summarized the anticancer efficacy of *β*-lactam derivatives, carbazoles, isatins, PBDs, and pyrido[2,3-d]pyrimidine derivatives.

Cancer has become a leading cause of mortality in developed nations. It can be instigated by genetic mutations in DNA. By 2020, approximately 10 million cancer-related deaths were anticipated globally [1]. Around 25.0% of cancer cases are linked to carcinogenic diseases, such as hepatitis and human papillomavirus infections. The most common malignancies worldwide include lung cancer(approximately 2.2 million cases), breast cance (approximately 2.09 million cases), colorectal cancer (approximately 1.9 million cases), prostate cancer (approximately 1.28 million cases), skin cancer (approximately 1.04 million cases), and stomach cancer (approximately 1.04 million cases). Consequently, the global incidence of cancer is rising, imposing considerable emotional and financial strain on individuals and families [2].

Cancer cells can exhibit either rapid or slow division, as seen by Burkitt’s lymphoma and plasma tumor cells, respectively. Proliferation is an essential factor in the expansion of cancer cells, marked by a rapid rate compared to normal cells. Sulphur mustards were utilized as weapons in World War I, resulting in bone marrow suppression, and later applied for chemoprevention. In the last 3–5 decades, several additional types have emerged, including purine inhibitors, folate analogs, and pyrimidine inhibitors [3]. The choice of anti-cancer pharmaceuticals for treatment differed based on the cancer.

Heterocyclic compounds, originating from organic synthesis and medicinal chemistry, are regarded as a fundamental field of organic chemistry. Heterocyclic compounds are chemical structures where 1 carbon atom in a ring is replaced by an element like sulfur (S), nitrogen (N), or oxygen (O). The presence of modified atoms and the dimensions of the scaffold in the molecule influence its chemical and physical properties. Alterations to the heterocyclic ring structure of a chemical can influence its efficacy in reducing inflammation, combating bacterial infections, inhibiting tumor proliferation, fighting viruses, and preventing fungal infections [4, 5]. Heterocyclic compounds containing nitrogen are widely found in nature and play a fundamental role in the structure of many vital chemicals. These compounds are essential building blocks in pharmaceuticals, agrochemicals, and various bioactive molecules. These include vitamins, hormones, pigments, alkaloids, medicines, herbicides, and antibiotics, all of which rely on nitrogen in their structure [6–10]. Numerous naturally occurring nitrogen-containing chemicals are referred to as alkaloids. Examples are atropine, nicotine, morphine, thiamine, and caffeine.

β-lactam derivatives, carbazoles, pyrrolo-benzodiazepines (PBDs), isatin derivatives, and pyrido[2,3-d]pyrimidines play a crucial role in medicinal chemistry and drug development. They are commonly found in both natural products and synthetic compounds. These heterocycles exhibit many biological actions, including antibacterial, antibiotic, antiinflammatory, antidiabetic, analgesic, antitumor, and anticancer properties [11]. They have a broad spectrum of biological actions, including the capacity to combat cancer. The anticancer properties of these heterocycles are attributed to many mechanisms, including the inhibition of angiogenesis, induction of apoptosis, suppression of cell proliferation, disruption of signal transduction pathways, DNA damage and repair processes, and immunological regulation [12]. These heterocycles have been found to interact with multiple molecular targets associated with the proliferation and survival of cancer cells. Figure 1 depicts a select array of β-lactam derivatives, carbazoles, isatin derivatives, PBD, and pyrido[2,3-d]pyrimidine-based anticancer agents [13]. Motivated by the anticancer properties of β-lactam derivatives, carbazoles, isatin derivatives, PBDs, and pyrido[2,3-d]pyrimidine derivatives, a study will be compiled on the anticancer potential of these heterocycles. This chapter examines the anticancer activity of β-lactam derivatives, carbazoles, isatin derivatives, PBD, and pyrido[2,3-d]pyrimidine derivatives.

**Figure 1.**
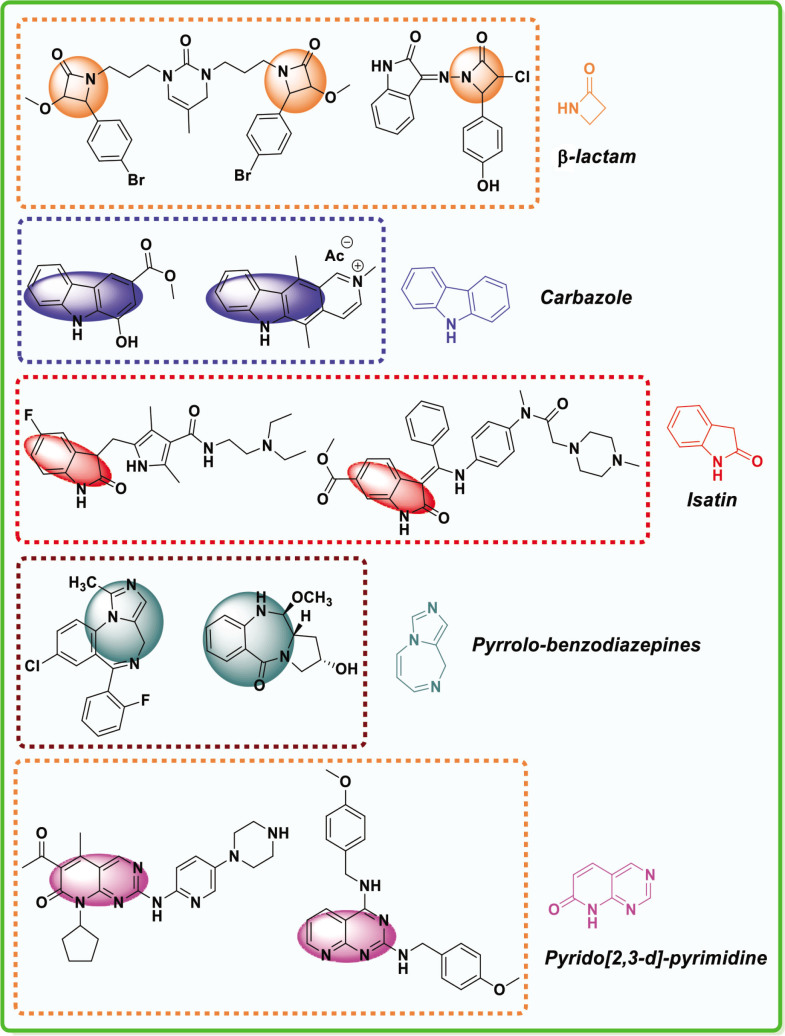
Selected anticancer derivatives of β-lactam derivatives, carbazoles, isatin derivatives, PBD, and pyrido[2,3-d]pyrimidine. PBDs, pyrrolo-benzodiazepines.

Fused heterocyclic compounds, such as β-lactam derivatives, carbazoles, isatin derivatives, PBD, and pyrido[2,3-d]pyrimidine derivatives, exhibit considerable promise as anticancer drugs. β-lactam derivatives, recognized for their antibacterial activities, also have lethal effects by inhibiting cancer cell proliferation. Carbazoles are flat, 3-ring molecules known for slipping between DNA strands and blocking topoisomerase enzymes, which leads to the death of tumor cells. Isatin derivatives serve as potent inhibitors of kinases and proteases, rendering them significant for targeting many cancer pathways. PBD demonstrates anticancer efficacy by binding to the DNA minor groove, resulting in DNA damage and cell cycle arrest. Pyrido[2,3-d]pyrimidine derivatives similarly target tyrosine kinases, essential elements of cancer cell signaling pathways, providing selective cytotoxicity towards malignant cells. These molecules, via various methods, underscore the adaptability of fused heterocycles in formulating targeted and efficacious cancer treatments.

## β-Lactam derivatives

**Figure 2.**
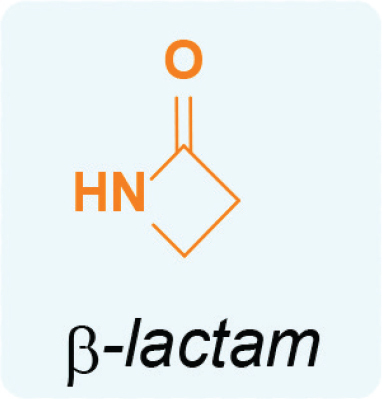
Structure of β-lactam.

β-Lactam antibiotics have a β-lactam ring inside their molecular architecture. The β-lactam is a cyclic amide classified as a 4-membered lactam. This specific ring is essential to the antibiotic efficacy of these compounds, as demonstrated in Figure 2.

β-Lactams are bioactive compounds with a diverse range of therapeutic properties, including antifungal, antibacterial, anti-inflammatory, cholesterol absorption inhibition, antihepatitic, antihyperglycemic, and analgesic effects [14–23]. A considerable focus has been directed towards the anticancer properties of β-lactam worldwide [24, 25]. In the past decade, various research groups have undertaken extensive studies on the synthesis [26] and anticancer characteristics [27] of β-lactam derivative molecules. The β-lactam chainbased anticancer drug candidate is depicted in Figure 1. Due to their diverse pharmacological actions, β-lactam derivatives are appealing candidates for drug discovery because of their versatility and allure. Moreover, several of these compounds exhibit significant efficacy against cancer cell lines, rendering them very compelling. The β-lactam molecule offers a substantial foundation for the development of new anticancer drugs [28]. Fungal species from the Penicillium genus produce β-lactam compounds. In 1928, Sir Alexander Fleming discovered one such compound and named it “penicillin” while working at St. Mary’s Hospital in London, England [29]. Fleming observed the phenomenon of bacteriolysis occurring in a broth tainted with Penicillium. β-lactam, also known as azetidin-2-one, is an important molecule used to make β-amino ketones, γ-amino alcohols, and other useful compounds. It belongs to a group called lactams, which are ring-shaped amides. In β-lactams, the nitrogen is bonded to the β-carbon next to a carbonyl group, as shown in Figure 1. β-Lactam antibiotics are widely used to treat bacterial infections. Moreover, new families of β-lactams have demonstrated anticancer effects. N-thiolated β-lactams selectively trigger death in cancer cells by DNA damage, while exhibiting no effect on normal cells [30, 31]. The identification of polyaromatic chemicals has revealed their capacity to inhibit or retard the proliferation of cancer cells. Conversely, 4-alkylidene β-lactam compounds could inhibit the activity of matrix metalloproteinases and leukocyte selections.

## Function of β-lactam as prodrug in anticancer chemotherapy

**Figure 3.**
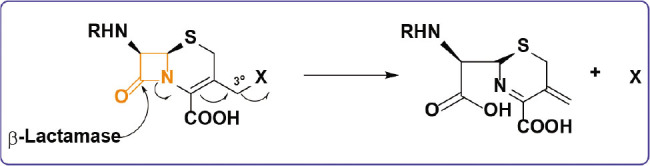
Synthesis of cephalosporin prodrug.

β-lactams have significant efficacy and possess minimal toxicity, rendering them a compelling focus for pharmaceutical development. In the domain of prodrugs, β-lactams have been shown to be beneficial in facilitating the targeted delivery of chemotherapeutic agents to specific tumor sites. Investigations have been undertaken to examine the potential of β-lactams as prodrugs for anticancer chemotherapies, offering numerous benefits. Targeted delivery enables the specific release of active medication into cancer cells, hence minimizing the systemic toxicity of the treatment. Moreover, β-lactam prodrugs can enhance the solubility and stability of anticancer agents with low solubility. They can diminish resistance by leveraging the augmented synthesis of β-lactam in cancer cells. Prodrugs possess the capability to circumvent resistance mechanisms. Due to their capacity to facilitate pharmaceutical release and absorption, β-lactam prodrugs possess the potential to improve medicine efficacy while concurrently diminishing the incidence of adverse effects. In prodrug-based cancer therapy, cephalosporins have been the most employed β-lactam. The inherent reactivity of cephalosporins made them suitable candidates for the prodrug category. The enzyme β-lactamase catalyzes the hydrolysis of the β-lactam ring, thereby triggering a process that releases the anticancer medication in the third position, as shown in Figure 3

Recent +years have witnessed considerable interest in utilizing cephalosporins for the targeted delivery of anticancer agents to tumor cells via antibody-directed enzyme prodrug therapy (ADEPT) [32, 33]. The objective of this therapy was to mitigate the detrimental effects of chemotherapy and augment its efficacy by delivering the toxic agent directly to the tumor sites. This can be accomplished by employing an antibody that specifically targets an antigen associated with the tumor. The antibody will facilitate the delivery of an enzyme to the tumor sites. After the enzyme has been removed from the blood-stream, a low-toxicity prodrug will be delivered. Inside the tumor, the prodrug would be subjected to enzymatic catalysis, resulting in the release of a highly poisonous agent. The rising annual influx of new patients has necessitated a heightened focus on medical research to develop suitable chemotherapeutic agents. β-lactam chemicals can function as prodrugs that specifically target neoplastic cells.

## Isatin derivatives

**Figure 4.**
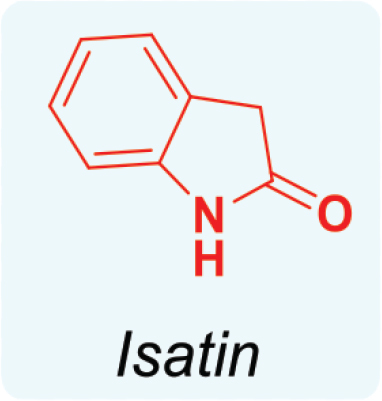
Structure of isatin.

Isatin is an organic compound with the formula C_8_H_5_NO_2_, derived from indole. It contains a fused benzene and pyrrole ring, known as the indole core, and has a keto group at position 2 and a carbonyl group at position 3, as shown in Figure 4.

**Figure 5.**
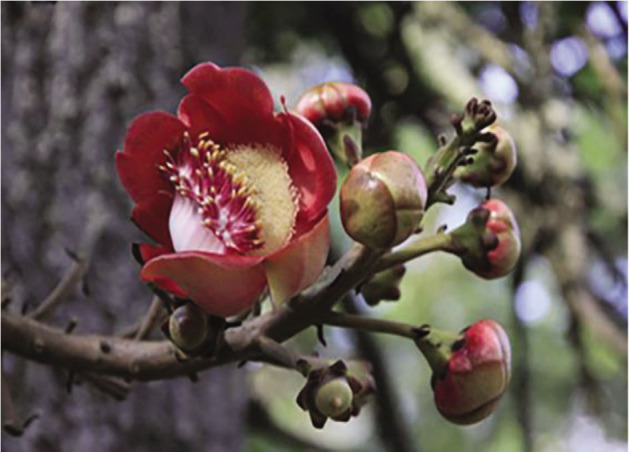
An image of *C. guianensis* (Abricó-de-macaco) tree.

Isatin, also called 1H-indole-2,3-dione, is a naturally occurring alkaloid. It appears as a reddish-orange powder and is mainly extracted from the fruits of *Couroupita guianensis*. It is also found in other plants such as *Melochia tomentosa, Isatis tinctoria*, and *Boronia koniamboensis* [34] (Figure 5). Isatin is a naturally occurring compound in humans that exhibits several biological effects, including inducing anxiety, sedation, and anticonvulsant properties. It also functions as a potent inhibitor of atrial natriuretic peptide receptors in laboratory tests. Isatin is produced in humans as a metabolic byproduct of tryptophan or epinephrine (adrenaline) metabolism. Endogenous isatin functions as a monoamine oxidase (MAO) inhibitor, specifically targeting the MAO-B isoform [35]. Isatin derivatives demonstrate cytotoxic effects on human cancer cell lines [35]. Additionally, its chemical derivatives show toxic effects against certain human cancer cells. Isatin, as a versatile chemical, acts as a precursor for several derivatives featuring the oxindole moiety. These compounds have a wide range of biological and pharmacological characteristics. Research has been documented regarding the formation [36] and possible applications [37–39] of this chemical.

**Figure 6.**
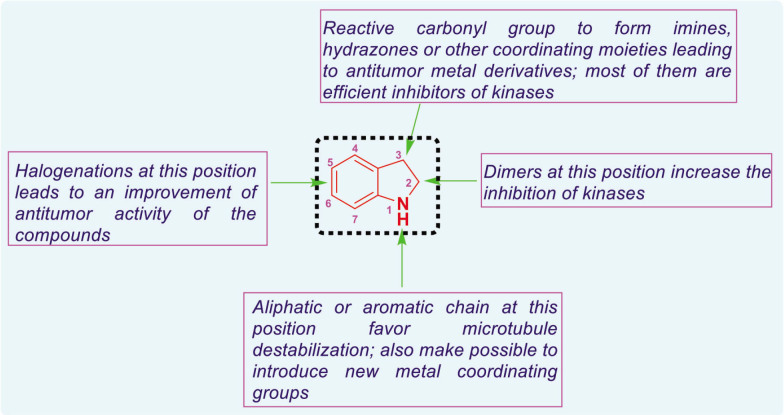
A structure–activity relationship of isatin derivatives [40].

Isatin is a multifaceted heterocyclic compound that demonstrates a broad spectrum of biological activities, comprising anticancer [41], anti-tubercular [42, 43], antimalarial [44, 45], anti-bacterial [46], anti-convulsant [47], and antiviral capabilities [48, 49]. The therapeutic applications of isatin, together with its varied pharmacological properties and several options for structural modification, have intrigued medicinal chemists, who are currently dedicated to enhancing the understanding and potential of this prolific heterocycle [40, 50]. Anticancer and antimicrobial drugs were extensively utilized in biological research. Isatin derivatives possess extensive applicability due to their inherent structural flexibility, facilitating the development of diverse frameworks tailored for specific biological or chemical properties of interest. Figure 6 depicts the structure-activity relationship of isatinderived anticancer agents.

## Anticancer activities of functionalized isatins

Isatin derivatives have many applications because they are very versatile, allowing the creation of different structures with specific reactivity or desired chemical properties.

Sunitinib and toceranib are 2 isatin-based drugs approved for cancer treatment. Other related drugs like nintedanib, orantinib, and semaxinib are currently being tested in clinical testing for their potential to fight cancer. Sunitinib reversibly binds to the adenosine triphosphate (ATP) binding sites of kinases, hence inhibiting their enzymatic activity in protein phosphorylation. It impedes the multiplication of cancer cells, resulting in their eventual elimination. Toceranib, akin to sunitinib, operates as a selective inhibitor of certain receptor tyrosine kinases (RTKs), resulting in the initiation of programmed cell death in neoplastic cells within a living organism. Toceranib phosphate is an orally bioavailable formulation of Sunitinib. Consequently, toceranib demonstrated both antiangiogenic and direct antitumor actions [51].

These intriguing chemicals have demonstrated the capacity to slow or cease tumor formation by affecting cellular growth, proliferation, survival, and migration. Nonetheless, various anticancer medicines exhibit adverse effects, including but not limited to diminished efficacy, diarrhea, hypertension, emesis, and vertigo.

## Isatin-derived amides and sulfonamides

The isatin compounds synthesized from sulfonamides show varying levels of efficacy against cancer treated with them induced apoptosis, diminished angiogenesis, and inhibited invasion. The reduction of BCl-2, activation of urokinase on plasminogen, and expression of heparanase were among the reported effects.

In 2017, Gao et al. [52] developed various compounds, including isatin as the principal constituent and o-phenylenediamine as the zinc-binding component. In the investigation of Eldehna [53], the investigators synthesized several amido/ureido-tethered isatin benzene sulfonamide hybrids and assessed their efficacy as inhibitors. Furthermore, Eldehna et al. [53] revealed the methodology for the synthesis and evaluation of a range of sulfonamides derived from indolinone. These sulfonamides block specific isoforms of carbonic anhydrase, particularly HCa IX and XII.

Wang et al. [54] synthesized severalα, β-unsaturated ketones generated from isatin in their investigation.They employed the (3-(4,5-dimethylthiazol-2-yl)-2,5-diphenyltetrazolium bromide) tetrazolium reduction (MTT) assay, a method utilizing 3-(4,5-dimethyl thiazole-2-yl)-2,5-diphenyl tetrazolium bromide, to assess the efficacy of these ketones in reducing cell proliferation across several cancer cell lines.

## Spiro compounds derived from isatins

Research on the synthesis of spirocyclic compounds from isatins began in the mid-20th century. The reaction of diazomethane with isatin initially produced a spirocyclic compound. The molecule, originating from isatin and incorporating a Michael acceptor, has been examined both in vitro and in vivo. It has been discovered to obstruct the activation of nuclear factor kappa B (NF-κB) mediated by IκB kinase β (IKKβ), which is crucial for the adaptive immune response initiated by tumor necrosis factor alpha (TNF) [55].

In 2015, Senwar et al. [56] developed a technique for synthesizing spirooxindole-derived morpholine-fused-1,2,3-triazoles. The procedure entailed the selective activation of isatin-spiro-epoxides by azide as a nucleophile, succeeded by sequential *o*-propargylation and intramolecular 1,3-dipolar cycloaddition. Isatin is an important structure in drug development because it is commonly found in biological systems and has a molecular makeup that supports many different biological activities. Isatin derivatives exhibit notable antiproliferative effects against several cancer cell types.

## Carbazoles

**Figure 7.**
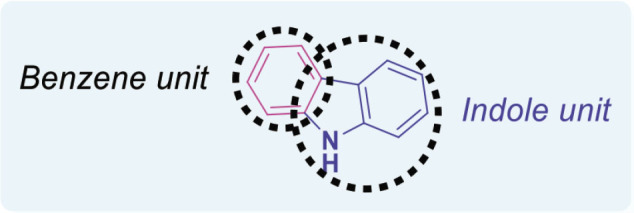
Structure of carbazole.

Carbazole is an aromatic compound made up of 2 benzene rings connected by a 5-membered ring that contains nitrogen. Its chemical formula is C_12_H_9_N, shown in Figure 7. Carbazole is important in organic chemistry and is widely used to make dyes, medicines, and organic semiconductors.

The carbazole moiety has been an essential structural component of physiologically active chemicals, whether natural or manufactured. Carbazole is crucial in the advancement of anticancer therapies, owing to its identification across diverse categories, such as bacteria, fungi, plants, and animals. The carbazole-based anticancer drugs were grouped according to their structures, starting from the basic 3-ring carbazole core and including more complex fused structures with 4, 5, 6, and 7 rings [57]. Carbazole compounds effectively stop the growth of many cancer cells by causing cell cycle arrest and triggering apoptosis (programmed cell death). However, certain anticancer pharmaceuticals are composed of carbazole-based compounds [58]. Figure 1 illustrates the carbazole-derived anticancer pharmaceuticals.

**Figure 8.**
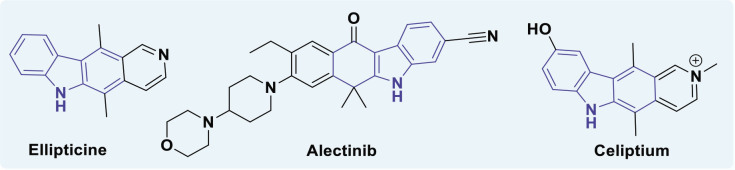
Structure of ellipticine, celiptium, and alectinib.

Carbazole is commonly found in the fruits, leaves, roots, and bark of the Rutaceae family. Carbazole may demonstrate anticancer properties by intercalating DNA, disrupting topoisomerases and telomerases, inhibiting kinase activation, or antagonizing estrogen receptors [59, 60]. In recent years, many carbazole compounds have shown promise as effective cancer treatments. Combining carbazole with different anticancer groups creates carbazole hybrids, which are more advanced and powerful. These hybrids can also target multiple cancer-related pathways at the same time. Carbazole hybrids can help reduce the strong side effects and drug resistance often seen with single drugs [61]. Numerous carbazole-derived compounds, including ellipticine, celiptium, and alectinib (Figure 8), have been authorized for cancer treatment [62]. Ellipticine operates by inhibiting DNA synthesis and inducing programmed cell death in malignant cells.

Ellipticine, identified in 1959, was first extracted from the foliage of *Ochrosia elliptica* (apocynaceae) prior to its complete synthesis. It may be considered the inaugural main compound of carbazole analogs. A derivative of ellipticine, known as N-methyl-9-hydroxyellipticinium acetate (eliptium), has been synthesized. Ceptium has been utilized for the treatment of metastatic breast cancer since 1982 [63]. Alectinib, an orally administered drug, received first approval from the US Food and Drug Administration (USFDA) in 2015 as the second agent to be authorized. Diaz et al. [34] have elucidated the anti-tumor efficacy of carbazole drugs, which specifically target microtubules and inhibit tubulin assembly.

## PBDs

**Figure 9.**
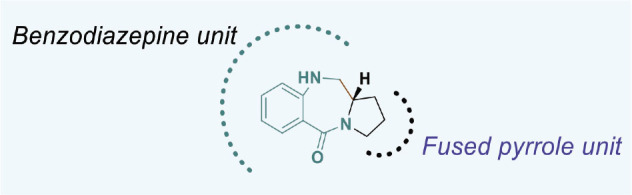
Structure of PBD core nucleus. PBDs, pyrrolo-benzodiazepines.

PBDs feature a distinctive chemical structure with a fused pyrrole and benzodiazepine ring system (Figure 9). The extensive structural diversity of PBDs facilitates the creation of compounds with properties tailored for specific applications. Researchers are diligently exploring modifications to improve the efficacy of anticancer therapies and reduce their adverse effects. PBDs are important in medicinal chemistry because their special structure allows them to bind DNA, making them promising for targeted cancer treatments.

**Figure 10.**
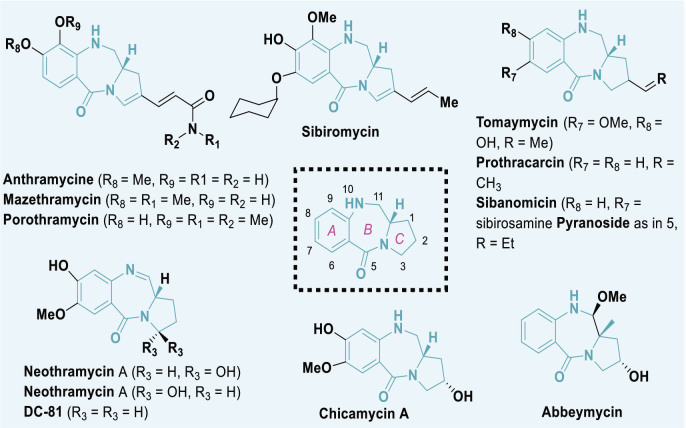
Few examples of PBD based biologically active compounds. PBDs, pyrrolo-benzodiazepines.

PBDs are a category of anthramycins, which are antitumor antibiotics that selectively bind to DNA. Anthramycin, a chemical sourced from *Streptomyces refuineus*, was initially identified in 1965 by Lei Mgruber and coworkers [64–66]. Subsequent compounds were obtained from several species of Streptomyces [67]. Prominent instances of these compounds encompass anthramycin [68, 69], mazethramycin [70], porothramycin [71], sibiromycin [72], tomaymycin [73], prothracarin [74], sibanomycin [75], neothramycins A and B [76], chicamycin A [77], and abbeymycin [78], as illustrated in Figure 10.

## Structure activity relationship

**Figure 11.**
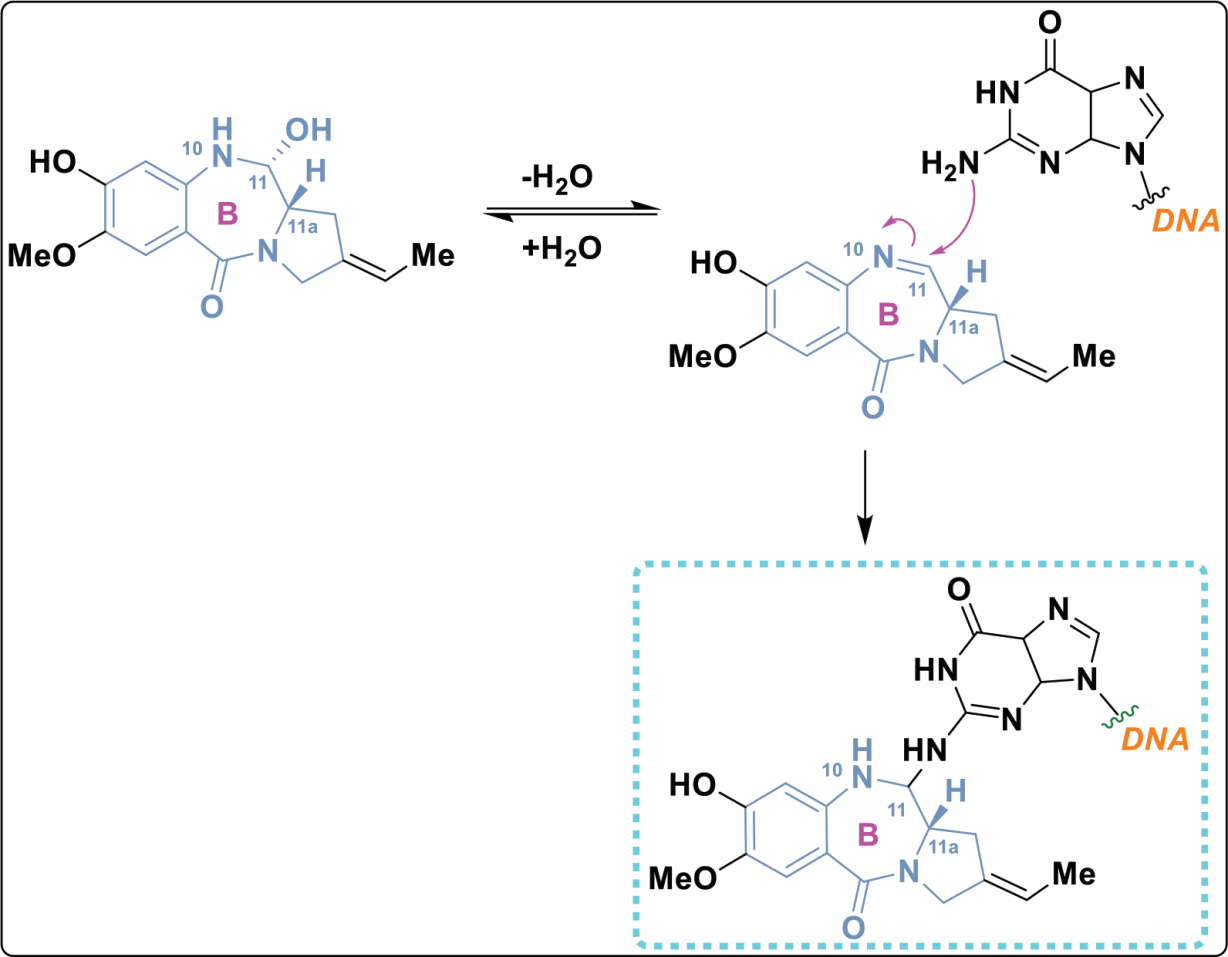
Binding mechanism of PBD with DNA. PBDs, pyrrolo-benzodiazepines.

The PBD structure can bind to DNA because a covalent bond forms between the N10 and C11 atoms in its core B ring(Figure 11) [79]. This bond interacts specifically with the N2 position of guanine bases in the minor groove of DNA [80]. The S-shaped bend at C11 gives the molecule a right-handed twist, allowing it to fit snugly into the slight curve of the DNA groove. This fit is important for the biological effects of molecule [81]. The primary methodologies employed to synthesize the PBD structure include the closure of the amino group and thioacetal to form a ring, the application of protected-NH ring closures, the cyclization of an azide and aldehyde, and cyclization reactions involving nitro reductions.

**Figure 12.**
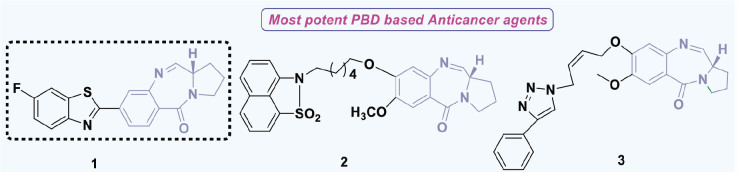
PBD derivatives (1-3) as anticancer agents. PBDs, pyrrolo-benzodiazepines. Findings from the literature review suggest that PBD compounds have been examined for their potential anticancer properties and have demonstrated encouraging results in inhibiting the advancement of cancer cells.

Findings from the literature review suggest that PBD compounds have been examined for their potential anticancer properties and have demonstrated encouraging results in inhibiting the advancement of cancer cells.

In 2012, Bose et al. [82] developed PBDs and assessed their cytotoxic efficacy against HL-60, THP-1, U-937, A-549, and Jurkat cell lines, which correspond to human promyelocytic leukemia, acute monocytic leukemia, lung cancer, and T-cell leukemia, respectively. Compound 1, shown in Figure 11, showed the strongest activity, with IC_50_ values ranging from 0.5 μM to 6.6 μM against the HL-60, THP-1, U-937, and Jurkat leukemia cell lines. These leukemia cell lines were grown in RPMI-1640 medium, while the A-549 cell line was grown in DMEM. For comparison, the standard drug Etoposide had IC_50_ values between 2.2 μM and 5.4 μM, showing its ability to inhibit various cancer cells.

In the same year, Kamal’s research group successfully developed PBDs linked to benzo-indolone molecules [83]. To evaluate the antiproliferative activity of these newly synthesized compounds, the MTT assay was conducted using a panel of human cancer cell lines derived from the skin (A431), lung (A549), prostate (PC-3), and colon (HT-29). The results demonstrated the potential of these compounds as anticancer agents. Compound 2 demonstrated the highest efficacy, with IC_50_ values of 1.2 μM for Colo-205, 1.7 μM for A431, 1.1 μM for PC-3, and 1.5 μM for PC-3, respectively. Domotopacin, the positive control, had IC50 values of 1.69 μM, 0.03 μM, 1.02 μM, and 2.51 μM.

In 2013, Chen et al. [84] effectively synthesized a novel series of PBD-triazole compounds with potent cytotoxic properties. The synthesized compounds demonstrated potent cytotoxicity against A375 cells. Compound 3 demonstrated a significantly greater inhibitory effect, evidenced by its IC_50_ value of 2.2 μM. The PBD-based heterocyclic compounds (1–3) (Figure 11) showed antitumor action. Compound 1 had the greatest efficacy among the compounds, with an IC_50_ value of 0.5 μM against THP-1 cells. This molecule includes a fluorine analog, which probably enhances its ability to penetrate cells. The presence of fluorine compounds typically increases cell permeability.

## Pyrido[2,3-d]pyrimidine

**Figure 13.**
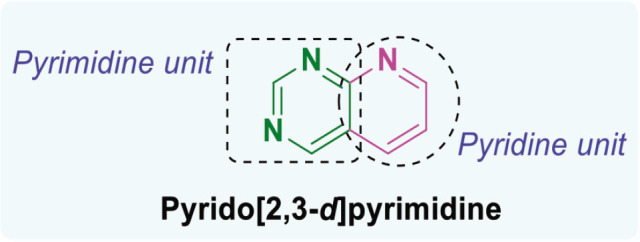
Structure of pyrido[2,3-d]pyrimidine framework.

Pyrido[2,3-d]pyrimidine is a heterocyclic compound with a fused ring structure that combines a pyridine ring and a pyrimidine ring (Figure 13). As bicyclic nitrogen-containing heterocycles, pyrido[2,3-d]pyrimidines are considered important scaffolds in medicinal chemistry. This structure is present in various biologically active compounds and pharmaceuticals, making it a valuable framework in drug development.

Pyridopyrimidines are ortho-fused bicyclic heterocycles formed through the fusion of pyridine and pyrimidine rings. 1,3,8-triazanaphthalenes constitute 1 of the 4 isomeric forms of pyridopyrimidines. These heterocyclic scaffolds are potential candidates for medicinal development due to their structural similarity to DNA bases [85]. Pyrido-pyrimidine is a prevalent heterocyclic structure capable of binding to multiple physiological receptors. Pyrido-pyrimidines have anticancer, antimicrobial, central nervous system depressant, anticonvulsant, antipyretic, and analgesic properties [86, 87]. Pyrido-pyrimidines function by blocking dihydrofolate reductase. Researchers are interested in pyrido-pyrimidine derivatives because of their diverse anticancer and antiproliferative effects [88].

In 2016, Naresh Kumar et al. [89] synthesized compounds by combining isoxazole/triazole with 7-(trifluoromethyl) pyrido[2,3-d]pyrimidine which were then evaluated for cytotoxicity against HeLa, PANC1, MDA-MB-231, and A549, cell lines. The compounds exhibited significant antitumor activity. Compounds 4 and 5 strongly suppressed the development of A549 and PANC-1 cells. Compound 4 exhibited GI_50_ values of 0.86 μM and 0.02 μM against A549 and PANC-1 cells, respectively. In contrast, compound 2 exhibited GI50 values of 0.03 M and 0.73 M against A549 and PANC-1 cells, respectively. The reference medication was Nocodazole, exhibiting IC_50_ values of 0.08 and 0.03.

Hou et al. [90] synthesized many new pyrido[2,3-d] pyrimidine derivatives and evaluated their efficacy against 5 human cancer cell lines: SK-BR-3, BT-474, MCF-7, A549, and MDA-MB-231. Pyrido-pyrimidine compounds exhibited potent anticancer properties. Compound 6 effectively suppressed cancer cell lines. The cell lines exhibited IC50 values ranging from 1.6 to 29.7. The values acquired were comparable to those of gefitinib, which exhibited IC_50_ values ranging from 0.5 μM to 37.8 μM against the identical cell lines.

Faidallah et al. [91] synthesized pyrido-pyrimidines and evaluated their effects on HepG2, MCF-7, and HT29 cell lines. A few of the compounds exhibited encouraging antitumor potential. Compounds 7, 8, and 9 exhibited comparable anticancer efficacy to doxorubicin, with LD_50_ values of 64.6 μM, 6.4 μM, and 25.2 μM for compound (7) 70.1 μM, 7.9 μM, and 28.8 μM for compound (8), and 71.2 μM, 8.91 μM, and 26.9 μM for compound (9). Conversely, doxorubicin had LD50 values of 3.0 μM, 4.0 μM, and 40.0 μM.

In 2017, Behalo and Mele [92] synthesized pyrido-pyrimidine derivatives and evaluated their efficacy as anticancer agents on PC-3 and MCF-7 cell lines. The identified compounds exhibited potent anticancer properties. The IC_50_ values of 9.5 and 10.3 for Compounds 10–11 exhibited the highest cytotoxicity against PC-3 cell lines. The reference medication 5-Fluorouracil exhibited IC50 values of 4.9 g/mL and 4.7 g/mL for PC-3 and MCF-7 cell lines, respectively.

In 2018, Banda et al. [93] synthesized pyridopyrimidine compounds and evaluated their efficacy against 4 human cancer cell lines: Colo205, B16F10, U937, and THP-1. Most derivatives eradicated all cells at concentrations <100 g/mL. Compound 12 exhibited the highest efficacy against B16F10 cells, demonstrating an IC50 of 19.0 g/mL. 5-fluorouracil, as a reference medication, exhibits IC50 values of 4.0 g/mL, 9.8 g/mL, 0.9 g/mL, and 0.5 g/mL against various cancer cell lines.

Substituted pyrido-pyrimidines were synthesized and evaluated in 2018 for their inhibitory effects on 5 tumor cell lines [10]. The compounds were powerful anticancer agents. Compound 13 exhibited significant anti-cancer effects on HCT-116, HepG-2, PC-3, and A549 cells, with IC50 values ranging from 0.3 μM to 9.6 μM. The reference drug Doxorubicin exhibited IC_50_ values ranging from 0.6 to 12.8.

In 2022, Al-Warhi et al. [94] synthesized and evaluated pyrido-pyrimidine compounds. Molecular docking was additionally investigated. Compound 14 exhibited comparable activity against MCF-7 and HepG2 cell lines, with IC50 values of 6.2 μM and 19.6 μM, respectively. Taxol, the control, has IC50 values of 8.5 μM and 14.6 μM, respectively.

**Figure 14.**
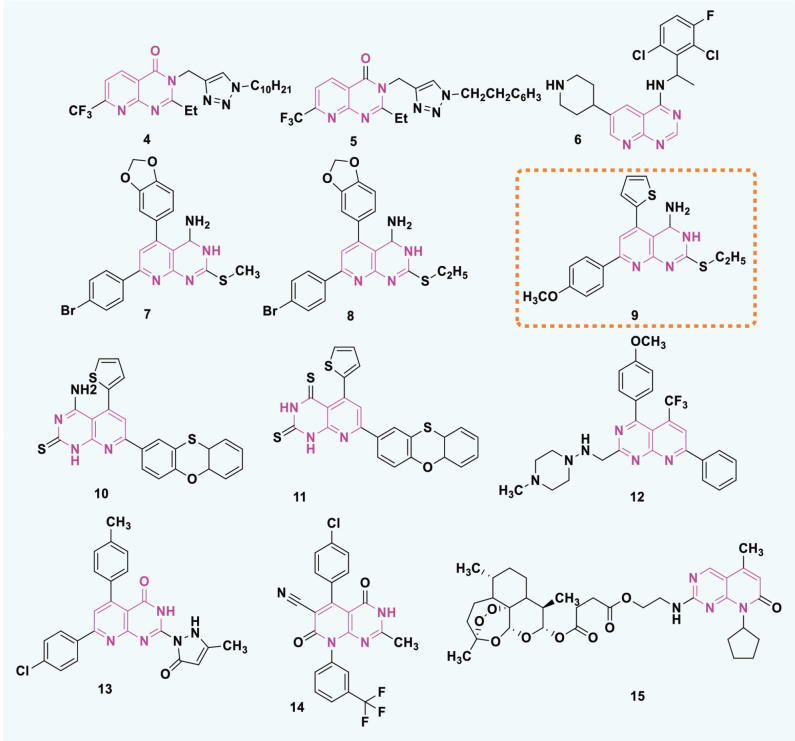
Examples of pyrido-pyrimidine based anticancer candidates.

Ding et al. [95] synthesized several pyrrolo(pyrido)-pyrimidine compounds utilizing DHA and ARS (Aminoacyl-tRNA synthetass). The compounds were subsequently evaluated for their capacity to suppress the proliferation of MDA-MB-436 and T-47D cells. The synthesized compounds 15 exhibited remarkable anticancer efficacy against T-47D, and MDA-MB-436 with IC50 values of 3.0 μM and 10.5 μM, respectively. Ribociclib, as a positive control, exhibits IC_50_ values of 24.4 M and 4.8 M. The pyrido-pyrimidine-based heterocyclic compounds (Figure 14) were identified as anticancer agents. Compound 9 exhibits the highest potency, with a GI50 value of 0.02 μM against PANC-1. The presence of an ethyl group at C-2 and a fluorine-substituted aromatic ring produces this effect. Table 1 represents summarized study of anticancer agents.

**Table 1.** Summary of anticancer agents

Type of compound	Compound name/structure	Activity	IC_50_	Type of cancer cell/mechanism of action)
*β* -Lactams	*N* -Thiolated *β* -lactams	Anticancer	-	DNA
	4-Alkylidene *β* -lactams	Anticancer	-	Matrix metalloproteinases and leukocyte selections
Isatins	Sunitinib	Anticancer	-	ATP binding sites of kinases
	Toceranib	Anticancer and antitumor	-	Inhibitor of certain RTKs
	Amido/ureido-tethered isatin benzene sulfonamide hybrids	Anticancer	-	Block specific isoforms of carbonic anhydrase, particularly HCa IX and XII
	α, β-Unsaturated ketones generated from isatin	Anticancer	-	Reducing cell proliferation across several cancer cell lines
	Spirooxindole-derived morpholine-fused-1,2,3-triazoles	Anticancer	-	Antiproliferative effects against several cancer cell types
Carbazoles	Ellipticine	Anticancer	-	Inhibiting DNA synthesis and inducing programmed cell death in malignant cells
	Ceptium	Anticancer	-	Treatment of metastatic breast cancer since
	Alectinib	Anticancer	-	Target microtubules and inhibit tubulin assembly
	Anthramycins	Antitumor antibiotics	-	Selectively bind to DNA
PBDs	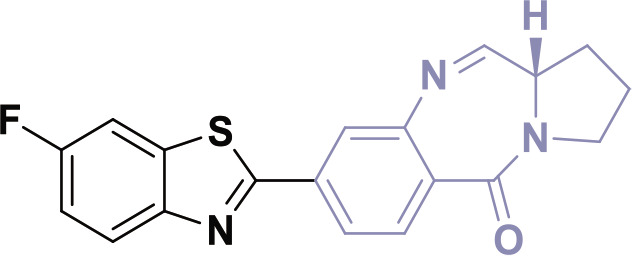	Anticancer	0.5–6.6 μM	HL-60, THP-1, U-937 and jurkat leukemia cell lines
	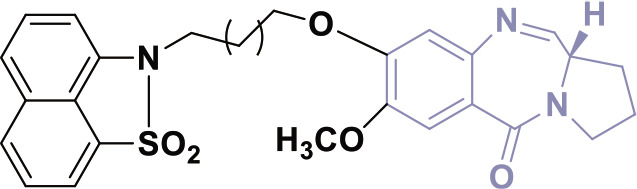	Anticancer	1.1–1.7 μM	Skin (A431), lung (A549), prostate (PC-3) and colon (HT-29)
	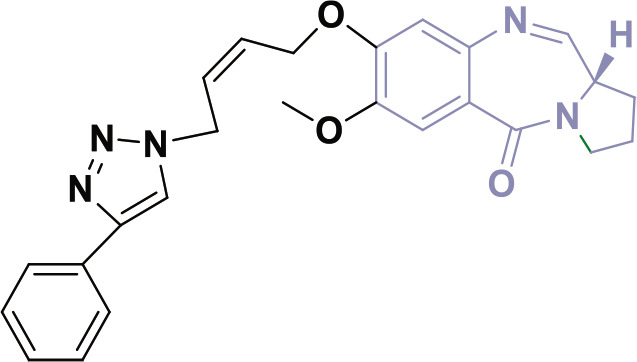	Anticancer	2.2 μM	Potent cytotoxicity against A375 cells
Pyrido[2,3-d] pyrimidines	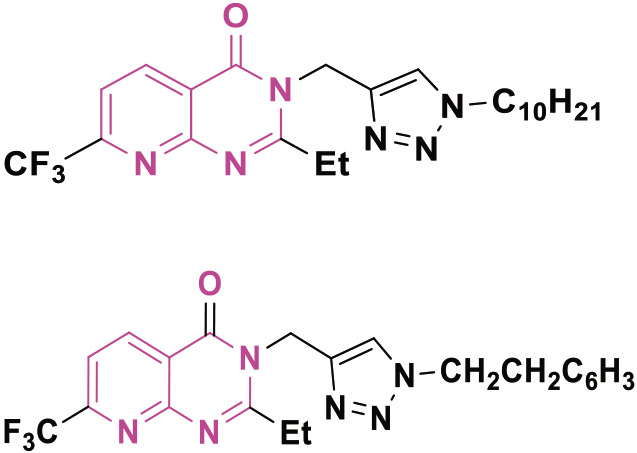	Anticancer	0.02–0.86 μM	Cytotoxicity against HeLa, PANC1, MDA-MB-231, and A549 cell lines
	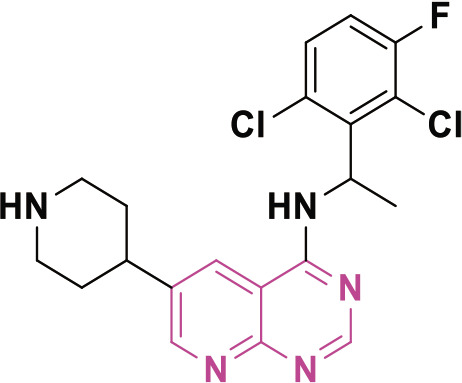	Anticancer	1.6–29.7 μM	SK-BR-3, BT-474, MCF-7, A549, and MDA-MB-231
	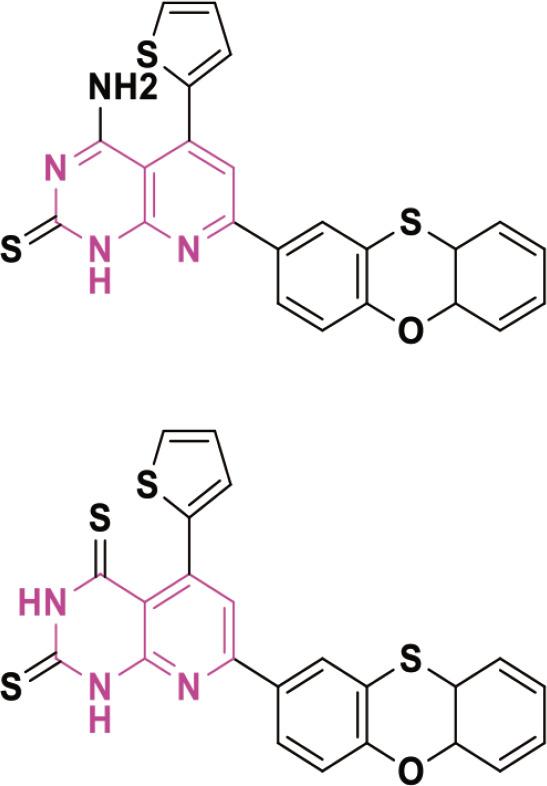	Anticancer	9.5–10.3 μM	Cytotoxicity against PC-3 cell lines
	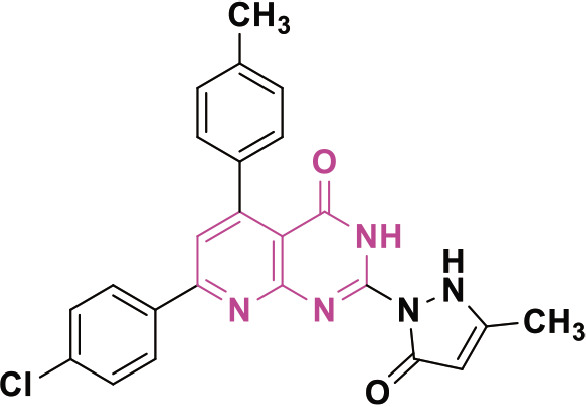	Anticancer	0.3–9.6 μM	HCT-116, HepG-2, PC-3, and A549
	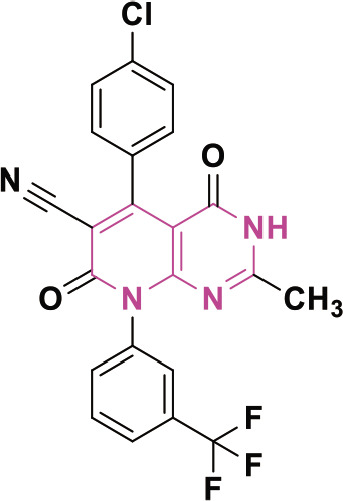	Anticancer	6.2 and 19.6 μM	MCF-7 and HepG2 cell lines
	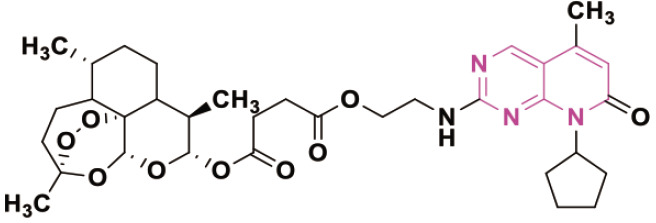	Anticancer	3.0 and 10.5 μM	T-47D and MDA-MB-436

1 ATP, adenosine triphosphate; PBDs, pyrrolo-benzodiazepines; RTKs, receptor tyrosine kinases.

## Conclusions

In summary, many fused heterocyclic compounds, such as carbazoles, β-lactam derivatives, isatin derivatives, PBDs, and pyrido[2,3-d]pyrimidine derivatives, exhibit considerable potential as anticancer drugs. Multiple research teams globally have investigated the anticancer efficacy of these compounds, with variants demonstrating substantial activity and a broad spectrum of IC_50_ and GD_50_ values against numerous cancer cell lines, including HL-60, THP-1, HeLa, U-937, Jurkat, A-549, PANC1, and MDA MB-231. These chemicals have demonstrated satisfactory to fascinating efficacy in inhibiting malignant cell lines, frequently in comparison with reference medications such as etoposide, doxorubicin, nocodazole, ribociclib, gefitinib, and 5-Fluorouracil. The anticancer efficacy of these chemicals is ascribed to many mechanisms, including enzyme inhibition, DNA intercalation, and disruption of cancer cell signaling pathways. Their structural intricacy and functional adaptability offer distinct benefits in targeting cancer types, surmounting medication resistance, and minimizing off-target consequences. Ongoing investigation and refinement of these heterocyclic frameworks *via* synthetic alterations and structure-activity relationship analyses will further improve their medicinal efficacy and safety profiles. Subsequent study must concentrate on elucidating their pharmacokinetics, toxicity, and clinical relevance. The use of modern methodologies, including molecular docking, high-throughput screening, and targeted drug delivery systems, indicates that these molecules have significant potential for advancing innovative and effective anticancer therapeutics.

This review thoroughly encapsulated the anticancer activity of β-lactams, carbazoles, isatins, PBDs, and pyrido[2,3-d] pyrimidines, offering valuable insights into their potential as chemotherapeutic agents. Despite the promising preclinical data, the translational journey of these scaffolds into clinical applications remains in its nascent stages. The β-lactams, carbazoles, isatins, PBDs, and pyrido[2,3-d]pyrimidines scaffolds represent a fertile ground for anticancer drug discovery. Their continued investigation, integration with emerging technologies, and multidisciplinary research approaches are vital to unlocking their full therapeutic promise.
